# Predicting the severity of dengue fever in children on admission based on clinical features and laboratory indicators: application of classification tree analysis

**DOI:** 10.1186/s12887-018-1078-y

**Published:** 2018-03-13

**Authors:** Khansoudaphone Phakhounthong, Pimwadee Chaovalit, Podjanee Jittamala, Stuart D. Blacksell, Michael J. Carter, Paul Turner, Kheng Chheng, Soeung Sona, Varun Kumar, Nicholas P. J. Day, Lisa J. White, Wirichada Pan-ngum

**Affiliations:** 1 Department of Tropical Hygiene (Biomedical and Health Informatics), Faculty of Tropical Medicine, Mahidol University, Bangkok, Thailand; 2 National Electronics and Computer Technology Center (NECTEC), Bangkok, Thailand; 3 Mahidol-Oxford Tropical Medicine Research Unit, Faculty of Tropical Medicine, Mahidol University, Bangkok, Thailand; 4 Centre for Tropical Medicine and Global Health, Nuffield Department of Medicine, University of Oxford, Oxford, UK; 5 Institute of Child Health, University College London, London, UK; 6 Angkor Hospital for Children, Siem Reap, Cambodia

**Keywords:** Classification tree, Dengue, Severity, Cambodia, Data mining, Children

## Abstract

**Background:**

Dengue fever is a re-emerging viral disease commonly occurring in tropical and subtropical areas. The clinical features and abnormal laboratory test results of dengue infection are similar to those of other febrile illnesses; hence, its accurate and timely diagnosis for providing appropriate treatment is difficult. Delayed diagnosis may be associated with inappropriate treatment and higher risk of death. Early and correct diagnosis can help improve case management and optimise the use of resources such as hospital staff, beds, and intensive care equipment. The goal of this study was to develop a predictive model to characterise dengue severity based on early clinical and laboratory indicators using data mining and statistical tools.

**Methods:**

We retrieved data from a study of febrile illness in children at Angkor Hospital for Children, Cambodia. Of 1225 febrile episodes recorded, 198 patients were confirmed to have dengue. A classification and regression tree (CART) was used to construct a predictive decision tree for severe dengue, while logistic regression analysis was used to independently quantify the significance of each parameter in the decision tree.

**Results:**

A decision tree algorithm using haematocrit, Glasgow Coma Score, urine protein, creatinine, and platelet count predicted severe dengue with a sensitivity, specificity, and accuracy of 60.5%, 65% and 64.1%, respectively.

**Conclusions:**

The decision tree we describe, using five simple clinical and laboratory indicators, can be used to predict severe cases of dengue among paediatric patients on admission. This algorithm is potentially useful for guiding a patient-monitoring plan and outpatient management of fever in resource-poor settings.

## Background

Dengue fever causes a high burden of disease and mortality across tropical and subtropical regions in Southeast Asia, Africa, the Western Pacific, and the Americas [1]. Dengue virus comprises five serotypes, DENV-1, DENV-2, DENV-3, DENV-4 and DENV-5, which are transmitted by *Aedes aegypti* mosquitoes [2–4]. An estimated 2.5 billion people worldwide are at risk of dengue. More than 50 million dengue infections are estimated to occur annually, of which approximately 500,000 result in hospital admissions for severe dengue in the form of dengue haemorrhagic fever (DHF) or dengue shock syndrome (DSS), principally among children [5].

Dengue infection is frequently confounded with other febrile illnesses (OFI), presenting with non-specific clinical symptoms and clinical features analogous to OFI. During the early stages of dengue, the presence of non-specific febrile illness makes precise diagnosis strikingly difficult, resulting in inefficient treatment and possible increases in morbidity and mortality [2, 6]. Severe dengue fever, if not appropriately managed, may lead to rapid death, particularly in children [7, 8]. In addition, the lack of necessary laboratory facilities, particularly in remote, rural areas, may cause difficultly in discriminating dengue infection from OFI [9]. Dengue is one of the most common vector-borne diseases in Southeast Asia, and one of the most important mosquito-borne viral diseases with an epidemic potential in the world [10].

Dengue was first included in Cambodia’s national surveillance programme in 1980. Since 2000, between 10,000 and 40,000 dengue cases have been reported annually by the Dengue National Control Program [11], from a total population of approximately 13.5 million people [12]. The true incidence of the burden of disease in Cambodia remains under-reported due to difficulties in diagnosing dengue infection, especially in hospitals [13]. In this study, data from a cohort of children that were admitted with febrile illness to Angkor Hospital for Children, Siem Reap, Cambodia, during a one-year period were retrospectively analysed using a data mining approach. This approach used a classification and regression tree, or CART, which was first introduced by Breiman et al. [14]. This is a common tool used in data mining, which creates a model or algorithm that predicts the value of a target variable based on several input variables. In our study, CART was constructed to predict the severity of dengue infection based on early clinical and laboratory indicators. The model was then evaluated against the final diagnoses.

## Methods

### Study design and data

We conducted a retrospective study of data derived from an investigation of febrile illness in children (“the fever study”) at Angkor Hospital for Children, Cambodia (AHC) [15]. This is a 70-bed children’s hospital in Siem Reap province, Cambodia, which provides free, comprehensive healthcare to children aged less than 16 years of age, and includes specialised medical and surgical inpatient and outpatient care. For the fever study, the inclusion criteria were age < 16 years, documented axillary temperature ≥ 38.0 °C within 48 h of admission, and informed consent by a parent or caregiver. Children who developed fever ≥ 48 h after admission or following surgery were excluded since they could be considered as having acquired a healthcare-associated infection [16, 17]. Integrated Management of Childhood Illness (IMCI) was used for the assessment and decision-making on whether to admit a patient to the hospital [18].

Data were collected on admission by clinicians using a specific case report form. Admission blood samples and, where possible, a convalescent serology sample taken on discharge, or seven days after admission, were taken for IgM antibody and NS1 antigen testing. All admitted patients were reviewed twice daily for eligibility and data collection quality. Data were collected between 12th October 2009 and 12th October 2010 from patients who were admitted to AHC.

Dengue diagnosis was based on the following laboratory diagnostic methods: 1) DENV NS1 antigen ELISA (Standard Diagnostics, Korea) to detect dengue-specific antigen in serum samples, 2) Panbio Japanese encephalitis virus (JEV) and dengue IgM Combo ELISA (Standard Diagnostics, Korea) was used to detect anti-JEV- and anti-DENV-specific IgM antibodies in serum samples, and 3) Dengue IgM capture ELISA (Venture Technologies, Malaysia) was used to detect anti-JEV- and anti-DENV-specific IgM antibodies in cerebrospinal fluid (CSF) specimens [15].

Patients were classified as having dengue virus infection if NS1 antigen was detected in their serum by ELISA, or if paired sera from acute and convalescent time points (≥7 days following the acute sample) showed rising or static anti-dengue IgM (and anti-dengue IgM was greater than anti-Japanese encephalitis IgM) [15]. NS1 antigen and IgM antibody results were combined in a Boolean manner using AND/OR operators to ensure that the entire temporal spectrum of patient presentations during the acute phase of dengue infection were covered, with NS1 antigen present in the serum in the early phase of infection and dengue IgM antibodies usually present after 2–5 days of infection [19]. The ratio of anti-dengue to anti-JEV IgM levels was used to determine if the infection was dengue or Japanese encephalitis virus, whose antibodies often have some cross-reactivity when co-circulating in the same area. Children less than 60 days old were not tested for dengue virus infection.

All confirmed dengue cases were further categorised as either severe or non-severe dengue. From our literature review we noted that, although the revised 2009 WHO classification was said to be an improvement on the 1997 WHO classification, there was still a need for training, dissemination of relevant information, and further research on the warning signs of severe dengue [20]. The classification was also considered by many to be too broad, requiring more specific definitions of the warning signs [21], that it increased the workload for health care personnel, and was not simple or user-friendly enough [22]. In our study we categorised dengue cases as severe based on a two–step process. The first step was to take into account all confirmed dengue cases with intensive care unit (ICU) admission, together with the 2009 WHO dengue classification. Secondly, two independent paediatricians’ assessments were considered to a) exclude any ICU-admitted cases that might not have had severe dengue as their primary diagnosis and b) to include any non-ICU-admitted cases which may have actually presented with severe dengue but were not admitted for some reason, usually because of resource limitations. Grading the disease severity in these patients was challenging because only the early clinical presentation of dengue and limited laboratory indicators were available on admission i.e. the first recorded haematocrit, platelet counts, white blood cell (WBC) counts, urea, creatinine, and alanine aminotransferase (ALT) results, and the presence of urinary protein or red blood cells (RBC). The results of chest X-rays were not available to evaluate pleural effusion, nor were results of abdominal ultrasound available for the detection of peritoneal fluid (ascites). The presence of bleeding was not assessed other than by examining stool samples for blood. The case-by-case assessment and verification by the two clinicians was used as the reference for the predictive model.

### Data analysis and construction of a predictive model

The demographics and clinical characteristics of severe and non-severe dengue cases were described using the mean ± standard deviation (SD) if the data were normally distributed, or by median and range otherwise. Comparisons between the two groups were performed using the Student’s *t*-test for continuous variables if the data were normally distributed, otherwise the Mann–Whitney *U* test was used. A chi-square test was used for categorical data. A *p* value < 0.05 was considered significant. A classification and regression tree (CART) was constructed for predicting the severity of dengue cases based on their early clinical features and laboratory indicators on admission. The J48 algorithm was used for generating decision trees because it is able to handle nominal, categorical and numerical data, as well as missing values. The loss matrix was specified to differently weight misdiagnoses. In this study we assigned five times greater weight to false negatives when compared with false positives, i.e. the cost of misdiagnosing a patient with severe dengue was five times greater than a non-severe case being misdiagnosed as severe. Pruning and tuning parameters were applied to optimise the predictive model by avoiding an over-complex tree, and thus increasing the model’s accuracy. The 10-fold cross-validation function provided by Weka was used to estimate the out-of-sample accuracy, given the constraint on data availability and avoiding the over-fitting issue. Put simply, it split the data set into ten partitions, nine of which were for training, with one partition for testing. The tree model was built on the training set, and applied to the testing set. To reduce variability, multiple rounds of cross-validation were performed using different partitions, and the validation results were combined over the rounds to estimate the model’s performance [23].

Once the final tree was obtained, the significance of each predictive factor was then quantified through multiple logistic regression with the ‘enter’ method of selection (i.e. all variables were included in the model) and reported as an odds ratio (OR) and 95% confidence interval (95% CI). Descriptive analysis and multiple logistic regression were performed using the Statistical Package for the Social Sciences (SPSS) software, version 18.0 (SPSS, Inc., Chicago, IL, USA), and the CART was constructed with Weka, version 3.6.10 (University of Waikato, New Zealand).

### Parameterisation

Before data mining algorithms can be used, a target data set must be assembled and pre-processed, which involves cleaning, removing, grouping, and transforming the data. There were 24 variables originally available for the analysis. However, three variables were excluded from the analysis i.e. tourniquet test result was with more than 15% missing data points where as pulse rate and respiratory rate were age-dependent parameters. The latter ones were excluded from the analysis since they would not be practical to refer to if presented in the final model. For other variables with fewer than 15% missing values, the missing values were imputed using a one-by-one single imputation approach. The advantage of the imputation method over the tree-based mining algorithm within CART [24] is that it separates the missing data problem from the prediction problem, allowing different predictive modelling methods to be applied to the imputed data set [25]. In our study, some missing values were imputed with a single value, including the mean value for some variables (number of days of fever, capillary refill time, Glasgow Coma Score, and urea result) and the median value for others (haematocrit, creatinine, ALT, respiratory rate of infant, urinary protein and RBC, and WBC, neutrophil, lymphocyte, and platelet counts).

## Results

There were 3225 patient admissions during the study year, of which 1361 (42.2%) met the inclusion criteria. Of these, 136 (10.0%) were not enrolled, leaving 1225 febrile episodes in 1180 children, with 1144 children having a single episode, 31 children having two episodes, one child having three episodes, and four children having four episodes. The patients were mainly diagnosed as having lower respiratory tract infection (38.3%), undifferentiated fever (25.5%), or diarrhoeal disease (19.5%) [15]. Out of 1180 enrolled children, there were 69 deaths, the causes of which were: clinical pneumonia with no organism/virus identified (12 cases, 27.5%), dengue virus infection (eleven cases, including one with co-existent melioidosis, two with co-existent scrub typhus, and four with co-existent clinical pneumonia, 15.9%), and melioidosis (four cases, 5.8%). 941 non-dengue episodes and 86 episodes with no samples available were excluded from this analysis. Further details can be found in the original report [15].

**Fig. 1:**
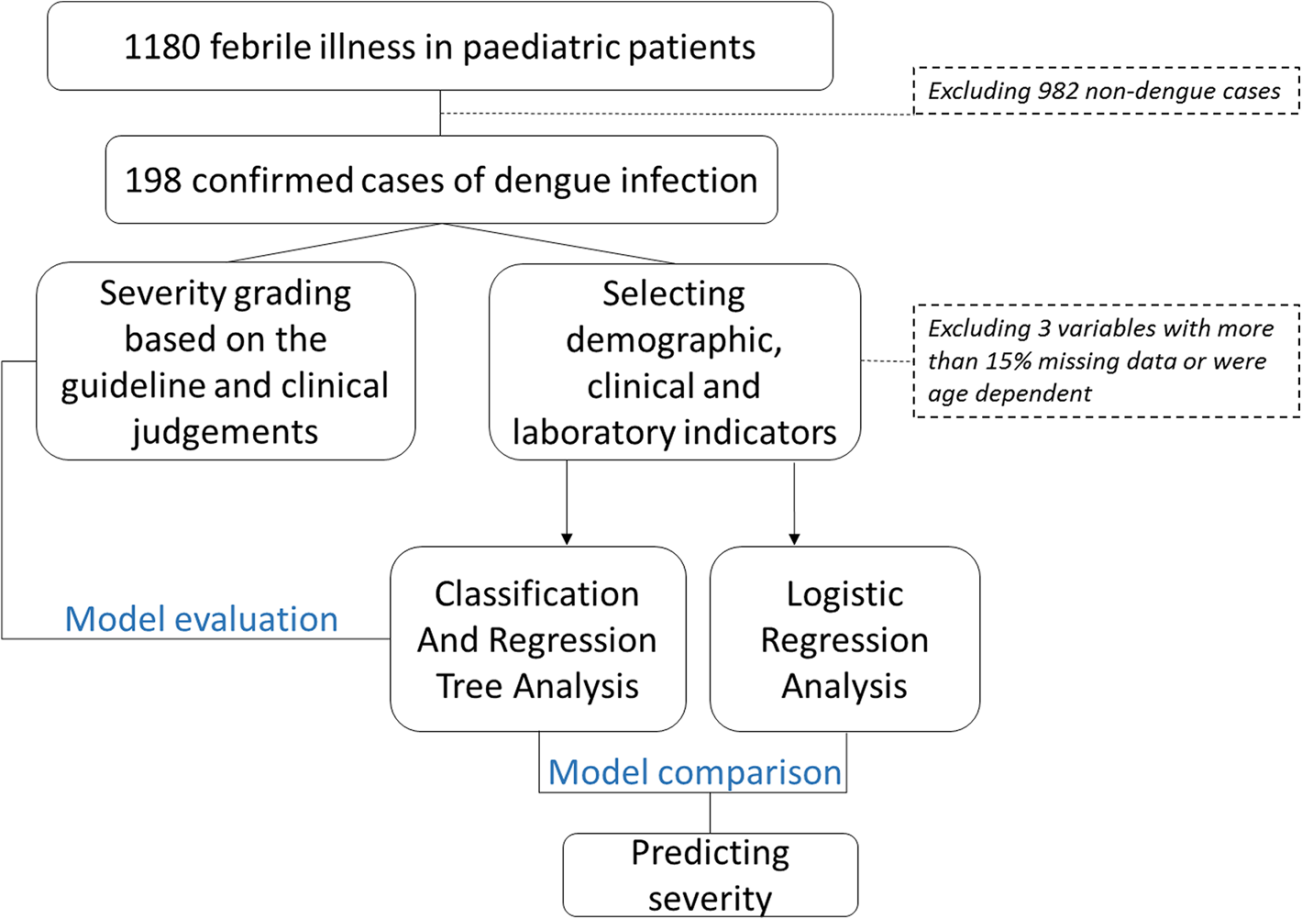
The flowchart of the study. Boxes show the total number of patients enrolled in the study, reasons for exclusion from the analysis, model construction and evaluation

Out of 198 confirmed dengue episodes, 43 episodes required ICU admission, with 29 of those classified as severe dengue based on their clinical signs, supported by two independent clinical opinions. Nine additional severe dengue episodes were included from non-ICU admissions, making a total of 38 episodes of severe dengue. There were eleven in-hospital deaths amongst all ICU-admitted patients with dengue virus infection, however dengue was the primary diagnosis in just five of these. Therefore, only these five cases were included in the severe dengue group. The flowchart of the study is shown in Fig. 1.

Clinical features, including blood in the stool, liver enlargement, ICU admission, number of days in ICU, low or high haematocrit, low or high WBC count, high creatinine, high urea, low platelet count, rapid pulse, rapid respiratory rate, low Glasgow Coma Score (GCS), pleural effusion (only one case), abdominal pain, urinary protein, urinary RBC, and high ALT, were considered on a case-by-case basis when clinicians classified dengue as severe or non-severe. The clinical features and laboratory indicators of the 38 severe dengue cases are shown in Table 1. The three most common features among patients with severe disease were ICU admission (76.3%), rapid respiratory rate (81.5%), and rapid pulse (65.7%). Severe dengue was more prevalent in children aged less than five years old. Vomiting and abdominal pain were significantly more common in the severe dengue group, as were rapid pulse and respiratory rate, increased capillary refill time, and low GCS. A significantly higher proportion of patients with severe dengue presented with a lower haematocrit, higher WBC and lymphocyte count, higher ALT level, together with the presence of urinary RBC (Table 2).

**Table 1:** Clinical features and laboratory indicators of 38 severe dengue cases based on the 2009 WHO dengue classification

Factor	N (%)
ICU admission	29 (76.3)
Average number of ICU days	4.6 days
Vomiting	15 (39.4)
Blood in stool	8 (21)
Rapid respiratory rate	31 (81.5)
Rapid pulse	25 (65.7)
Liver enlargement	22 (57.8)
Abdominal pain	9 (23.6)
Low WBC count	10 (26.3)
High WBC count	13 (34.2)
Low haematocrit	22 (57.8)
High haematocrit	3 (7.8)
Low platelet count	12 (31.5)
Low Glasgow Coma Scale	8 (21)
High urea	9 (23.6)
High creatinine	21 (55.2)
ALT > 1000	2 (5.2)
Urine protein > 100	5 (13.1)
Urine red blood cells	12 (31.5)
Death	5 (13)

**Table 2:** Clinical manifestations of 198 patients with dengue infection, including 38 with severe disease

Variables	Non-severe (*n* = 160)	Severe (*n* = 38)	*p* value
Demographics			
Male (n, %)	84 (52.5)	23 (60.5)	0.372
Age: 28 days to 1 year (n, %)	40 (25.0)	16 (42.1)	
≥ 1 year to < 5 years	45 (28.1)	14 (36.8)	0.012
≥ 5 years to < 16 years	75 (46.9)	8 (21.1)	
History/symptoms			
Number of days of fever	4.31	4.10	0.683
Vomiting (n, %)	94 (58.7)	15 (39.4)	0.032
Abdominal pain (n, %)	75 (46.8)	9 (23.6)	0.012
Headache or retro-orbital pain (n, %)	63 (39.3)	8 (21)	0.101
Clinical parameters			
Rash (n, %)	18 (11.2)	1 (2.6)	0.153
Temperature (°C)	38.76	38.63	0.234
Pulse/min	131.49	152.26	< 0.001
Capillary refill time	2.03	2.24	0.001
Respiratory rate	38.40	46.42	0.002
Liver enlargement (n, %)	64 (40.0)	22 (57.8)	0.127
Glasgow Coma Score	14.60	13.42	0.003
Laboratory parameters			
Haematocrit (%)	32.56	28.84	0.004
Platelets (per 10^3^/μl)	267.87	294.16	0.477
White blood cells (per10^3^/μl)	9.46	14.25	0.006
Neutrophils	5.64	8.01	0.46
Lymphocytes	2.76	5.14	< 0.001
Urea (mmol/L)	4.64	4.95	0.712
Creatinine (μmol/l)	68.29	68.71	0.932
Alanine transaminase, IU/l	78.99	177.08	0.002
Urine protein mg/dL	12.34	24.13	0.088
Urine red blood cells	2.84	10.32	0.009

**Fig. 2:**
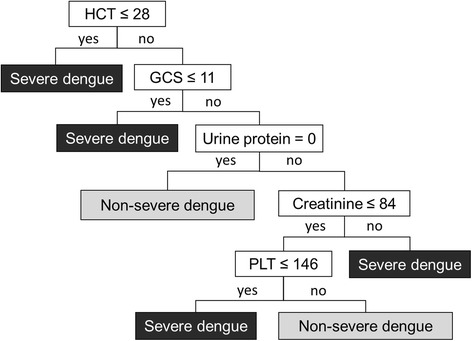
Clinical decision tree to distinguish severe dengue from all cases of dengue (HCT = haematocrit, GCS = Glasgow Coma Score, PLT = platelets)

The final decision tree algorithm included five clinical and laboratory parameters: haematocrit, GCS, urinary protein, creatinine, and platelet count. The sensitivity and specificity of the model were 60.5% and 65%, respectively (Fig. 2). The accuracy of the model was 64.1%, where the clinical diagnosis was used as the reference value. The area under the receiver operating characteristic (ROC) curve for logistic regression was 0.616. The final decision tree was then restructured using logistic regression analysis to estimate the impact of each CART-selected variable as represented by the OR and 95% CI.

Table 3 gives the estimated OR for each parameter selected by CART. Low haematocrit, low GCS, low platelet count, presence of urine protein, and high creatinine increased the probability of a diagnosis of severe dengue, with significant OR ranging from 1.47 to 13.73. The parameters that were statistically associated with severe dengue were 1) low haematocrit (OR = 7.114, 95% CI = 3.00–16.87, *p < 0*.001) and 2) low GCS (OR = 13.73, 95% CI = 3.46–54.50, *p* < 0.001). Although low platelet count (OR = 2.33, 95% CI = 0.95–5.76), presence of urine protein (OR = 1.83, 95% CI = 0.78–4.32) and increased serum creatinine (OR = 1.47, 95% CI = 0.51–4.25) were associated with an increased risk of severity, they were not shown to be statistically significant by regression analysis (Table 3).

**Table 3:** Output from logistic regression using the decision tree algorithm to predict severe dengue infection

Variable	OR	95% CI		*p* value
		Lower	Upper	
Haematocrit (> 28%)	1			
Haematocrit (≤ 28%)	7.114	3.000	16.869	< 0.001
Glasgow Coma Score (> 11)	1			
Glasgow Coma Score (≤ 11)	13.731	3.460	54.501	< 0.001
No urine protein	1			
Urine protein	1.832	0.777	4.319	0.167
Creatinine (≤ 84 μmol/l)	1			
Creatinine (> 84 μmol/l)	1.471	0.509	4.247	0.476
Platelets (> 146 × 10^3^/μl)	1			
Platelets (≤ 146 × 10^3^/μl)	2.334	0.946	5.763	0.066

## Discussion

Using a data mining approach, we have developed an algorithm using both simple clinical manifestations and laboratory indicators to predict the severity of dengue during the early phase of the illness. The final algorithm for predicting severe dengue (Fig. 2) comprised six components in order of their significance. The most significant factor in predicting severe dengue was low haematocrit, followed by a GCS of 11 or below as the second split if haematocrit was greater than 28, the presence of urine protein and creatinine above 84 μmol/l as the third split if GCS was above 11, and finally a platelet count of 146,000 per mm^3^ or less as the final split, if the presence of urine protein and creatinine was below 84 μmol/l.

Comparing the algorithm we derived with those reported in previous studies, we found both similarities and differences. Potts et al. constructed decision algorithms for predicting dengue shock syndrome (DSS) or dengue with significant pleural effusion [26]. The algorithm achieved a high sensitivity of 97%. Both low haematocrit and platelet counts were also identified as predictive factors in their work, although the cut-off values used in our algorithm were more extreme, i.e. for haematocrit ≤ 28 vs. ≤ 40, and for platelet count ≤ 146,000 vs. ≤ 160,200. The mechanism by which thrombocytopaenia is caused by dengue virus is complex [27]. Previous studies suggest that the virus probably contributes to bone marrow suppression and platelet destruction [28, 29]. To meet the WHO guidelines for classifying patients with DHF, thrombocytopaenia (platelet count ≤ 100,000) is required. Srikiatkhachorn et al. demonstrated that thrombocytopaenia was related to dengue severity and that not all severe cases would have been classified as DHF according to the WHO criteria [30]. Although thrombocytopaenia suggests that dengue infection is severe, a low platelet count is also common among OFI such as malaria and scrub typhus [31]. The 1997 WHO definition of DHF stated that a low platelet count (≤ 100,000), together with an increased haematocrit of ≥ 20% above the baseline value, is indicative of plasma leakage. In contrast, our results and those of Potts et al. suggested a drop in haematocrit as a sign of severity, especially among patients with internal bleeding in areas such as the gastrointestinal tract [26]. Our results also suggested a more extreme haematocrit value compared with the previous study (28% vs 40%) [27]. Although Potts et al. identified WBC count and monocyte percentage as important, our analysis did not identify monocyte results as significant even when included in the decision tree algorithm. In addition, Potts et al. evaluated predictors for DSS and dengue with significant pleural effusion, whereas in our study severe dengue was differentiated based on clinical features and laboratory indicators.

Another recent study, by Tamibmaniam et al., used simple logistic regression and identified three parameters, including vomiting, pleural effusion, and low systolic blood pressure, to predict severe dengue based on the 2009 WHO criteria [32]. This study did not specifically focus on children and included only female patients. The sensitivity and specificity achieved in its decision algorithm were 81% and 54%, respectively. Of the three parameters they identified, vomiting was the only parameter available in our study, and although it initially appeared to be significant in the severe group, it was not selected for the final tree.

Despite using a similar approach to predict somewhat similar outcomes to the aforementioned studies, we identified additional parameters that related to the severity of dengue, including GCS, urine protein, and serum creatinine. There are a number of possible explanations for these differences, as outlined below.

GCS is used to measure the level of consciousness (mental status changes) [33]. In our results, the node with GCS ≤ 11 (considered to be moderate) in the model was significant. Rao et al. showed that patients with dengue encephalitis had a GCS of 7–8 and recommended intubation and mechanical ventilator support during their hospitalisation [34].

Previous studies in which urine protein was associated with DHF or DSS used the urine protein-to-creatinine ratio [35, 36], but we used only a urine dipstick for this measure. The presence of urine protein in severe dengue could be due to plasma leakage.

An increased serum creatinine level indicates kidney dysfunction. In patients with DHF, a mild increase in serum creatinine is common, in contrast to the higher levels seen in severe dengue cases. Our model showed that a serum creatinine level > 84 mmol/l (4.6 mg/dl) was associated with severe dengue, a value similar to that found in Thai paediatric patients with DHF, whose mean serum creatinine was 4.9 mg/dl. That analysis also showed that 24 of 25 patients with acute kidney injury (AKI) had DSS as a final diagnosis. Of the 25 patients with DHF-associated AKI, 16 (64%) died as a result of profound shock, together with other conditions such as liver failure, respiratory failure, and severe bleeding [37]. Studies in adults have reported an AKI incidence of 14.2% among dengue patients, and those with AKI saw significant morbidity and mortality, longer hospital stays, and poor renal outcomes [38]. Early diagnosis of dengue infection, known clinical and laboratory characteristics and risk factors together with early detection of AKI using appropriate criteria [39], and monitoring for warning signs of severe dengue, are essential if AKI and other complications are to be avoided [40].

Although the two sets of WHO criteria from 1997 and 2009 are still debatable in terms of their ability to appropriately differentiate dengue from OFI and to classify disease severity [20–22, 41, 42], the problem is compounded by a lack of key data in resource-poor settings, making it difficult to apply the criteria. For instance, we lacked information on clinical bleeding sites, and were only able to detect gastrointestinal bleeding based on stool examinations. In addition, there was a lack of information on blood pressure or narrow pulse pressure to indicate whether a patient was in shock [43], no data on restlessness suggestive of circulatory failure, and no chest X-ray results to evaluate pleural effusion or abdominal ultrasound to detect ascites, both of which are important for identifying plasma leakage. The 1997 and 2009 WHO dengue guidelines also include a tourniquet test as a diagnostic tool for dengue in the early febrile phrase. However, the tourniquet test has been shown to have low sensitivity for dengue diagnosis, such that a negative result does not exclude dengue infection [44–46]. The tourniquet test had not been performed for the majority of patients in our data set and was thus not included in the analysis.

Regarding the two approaches used in this study, CART versus the more conventional approach of logistic regression, some points are worth mentioning here. Firstly, our main focus was on building the decision tree model from CART analysis. CART is non-parametric, and can manipulate numerical data which may be highly skewed, multi-modal, ordinal or non-ordinal in structure. CART is not significantly impacted by outliers in the input variables. The output of CART in the form of a decision tree is easy to follow and gives some visual information on the hierarchical importance of the variables from the top to the bottom of the tree, although calculating the importance matrices of the predictors in CART is not straightforward. In this study, therefore, we quantified the importance of each decision tree predictor via the odds ratio as calculated by logistic regression. Secondly, the ways in which the decision boundaries are generated in the two approaches are different. While the logistic regression generates a single boundary, a decision tree essentially partitions the data space into half-spaces using axis-aligned linear decision boundaries, giving a non-linear decision boundary. Either approach may be more applicable depending on the setting. Finally, the model’s accuracy was measured in different ways for each of the two approaches. For the decision tree model, the out-of-sample accuracy was estimated via cross-validation, i.e. the 10-fold cross-validation function in Weka allowed us to conveniently perform the cross-validation and directly report the model’s accuracy. For the logistic regression, however, the model’s accuracy was estimated from the classification table, which showed the number of observed against predicted outcomes, using a default cut-off value of 0.5 for the predicted probability. For all of the above reasons, it would have been difficult to compare the relative merits of the two methods used in our study.

There were several limitations with regard to the dataset used for this study. Firstly, the data came from just one hospital, a further indicator of the poor resources in the Southeast Asia region where dengue is endemic. Secondly, due to the lack of IgG antibody results it was not possible to interpret whether cases were primary or secondary dengue infections. This information could potentially be a useful early indicator for the severity of a dengue infection. Thirdly, the algorithm was derived from data collected within 48 h of admission among children aged less than 16 years old. If the model were to be used for older patients or in different regions, some adjustments to it may be necessary.

Although the cohort of 198 patients with confirmed dengue was relatively small, with an even smaller subset of just 38 severe dengue cases, the simple model we derived is still likely to be useful because it includes a small number of predictive variables that would probably be available in similar settings. In addition, a previous study by Carter et al. showed that the DENV rapid diagnostic test (RDT) had low sensitivity for the diagnosis of dengue infection [47]. However, the development of diagnostic tests for dengue has advanced rapidly. The NS1 test in particular has become widely available in many resource-limited settings. It is simple to use and has acceptable accuracy. If rapid diagnosis of dengue using NS1 can be achieved, our algorithm would prove very useful. This also highlights the importance of children attending for testing as soon as dengue is suspected, because NS1 detection is optimal during the first seven days of infection. The algorithm will become more relevant and useful as the rapid diagnosis of dengue becomes more common. By using our simple algorithm to help identify and predict severe dengue, we believe that there would be more room to focus on other, more serious bacterial diseases, which are all-too-common in these types of resource-poor settings.

## Conclusions

Our decision tree algorithm using simple clinical and laboratory indicators has a moderate classification accuracy for predicting the development of severe dengue fever among paediatric patients with confirmed DENV infection. The model demonstrates the importance of haematocrit and platelet levels for monitoring the severity of dengue, as indicated by the WHO criteria and previous studies. Our algorithm offers simple indicators for severity, including haematocrit, GCS, urine protein, creatinine, and platelet count, all of which are measured on admission. This model is potentially useful for guiding inpatient monitoring and outpatient management of fever cases. The model does require further validation against other datasets from cohort studies conducted in a variety of settings, with the goal of establishing a universal algorithm for guiding clinical management of severe dengue in resource-limited settings.

## Abbreviations

AHC: Angkor Hospital for Children
AKI: Acute kidney injury
ALT: Alanine aminotransferase
CART: Classification and regression tree
CI: Confidence interval
DHF: Dengue haemorrhagic fever
DSS: Dengue shock syndrome
GCS: Glasgow Coma Score
ICU: Intensive care unit
JEV: Japanese encephalitis virus
OFI: Other febrile illnesses
OR: Odds ratio
WBC: White blood cells
